# Synthesis and enzymatic ketonization of the 5-(halo)-2-hydroxymuconates and 5-(halo)-2-hydroxy-2,4-pentadienoates

**DOI:** 10.3762/bjoc.13.101

**Published:** 2017-05-26

**Authors:** Tyler M M Stack, William H Johnson Jr., Christian P Whitman

**Affiliations:** 1 Department of Molecular Biosciences, College of Natural Sciences, 1 University Station, University of Texas, Austin, TX 78712, USA; 2 Division of Chemical Biology and Medicinal Chemistry, College of Pharmacy, 1 University Station, University of Texas, Austin, TX 78712, USA

**Keywords:** dienol, enzyme kinetics, fluoride, halogen

## Abstract

5-Halo-2-hydroxymuconates and 5-halo-2-hydroxy-2,4-pentadienoates are stable dienols that are proposed intermediates in bacterial *meta*-fission pathways for the degradation of halogenated aromatic compounds. The presence of the halogen raises questions about how the bulk and/or electronegativity of these substrates would affect enzyme catalysis or whether some pathway enzymes have evolved to accommodate it. To address these questions, 5-halo-2-hydroxymuconates and 5-halo-2-hydroxy-2,4-pentadienoates (5-halo = Cl, Br, F) were synthesized and a preliminary analysis of their enzymatic properties carried out. In aqueous buffer, 5-halo-2-hydroxy-2,4-pentadienoates rapidly equilibrate with the β,γ-unsaturated ketones. For the 5-chloro and 5-bromo derivatives, a slower conversion to the α,β-isomers follows. There is no detectable formation of the α,β-isomer for the 5-fluoro derivative. Kinetic parameters were also obtained for both sets of compounds in the presence of 4-oxalocrotonate tautomerase (4-OT) from *Pseudomonas putida* mt-2 and *Leptothrix cholodnii* SP-6. For 5-halo-2-hydroxymuconates, there are no major differences in the kinetic parameters for the two enzymes (following the formation of the β,γ-unsaturated ketones). In contrast, the *L. cholodnii* SP-6 4-OT is ≈10-fold less efficient than the *P. putida* mt-2 4-OT in the formation of the β,γ-unsaturated ketones and the α,β-isomers from the 5-halo-2-hydroxy-2,4-pentadienoates. The implications of these findings are discussed. The availability of these compounds will facilitate future studies of the haloaromatic catabolic pathways.

## Introduction

Aromatic hydrocarbons and their halogenated derivatives are well known environmental contaminants [1–5]. Halogenated aromatic compounds are found in many industrial commodities such as pesticides, flame-retardants, hydraulic fluids, and synthetic intermediates for pharmaceutical agents [2–4]. Several strategies are being explored to remove these toxic compounds from the environment. One particularly attractive strategy is bioremediation, which uses microbial catabolic pathways to process the toxic species to metabolic intermediates that frequently can be channeled to the Krebs Cycle [2–5]. This approach requires a thorough understanding of each of the pathway steps. This information is also useful to predict the fate of halogenated species once released into the environment.

**Scheme 1:**
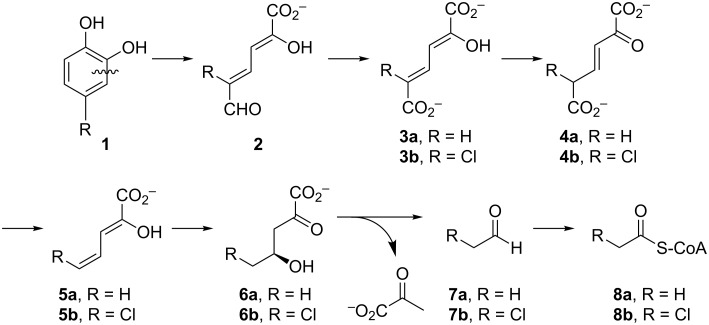
The *meta*-fission pathway in *P. putida* mt-2 and *Comamonas* sp. strain CNB-1. The degradation of toluene generates the intermediates where R = H (compounds **3a**–**8a**). The degradation of 4-chloronitrobenzene in *C.* sp. strain CNB-1 is proposed to use similar enzymatic steps where R = Cl (compounds **3b**–**8b**).

One major route for the degradation of aromatic compounds is the *meta*-fission pathway [6–7]. The enzymes and reactions of the *meta*-fission pathway in *Pseudomonas putida* mt-2 for monocyclic aromatic compounds (e.g., benzene, toluene, and alkyl-substituted derivatives) have been extensively studied for more than 60 years. Initially, the aromatic compound is converted to catechol or a catechol derivative. Subsequently, the resulting species undergoes *meta*-fission where this term refers to the position of the ring fission (shown on **1** in Scheme 1). An extradiol dioxygenase processes catechol **1** to 2-hydroxymuconate semialdehyde **2**, which is oxidized by an NAD^+^-dependent dehydrogenase to yield 2-hydroxymuconate (**3a**) [6–7]. Ketonization of **3a** to 2-oxo-3-hexenedioate (**4a**) is catalyzed by 4-oxalocrotonate tautomerase (4-OT) [8]. Decarboxylation of **4a** by the metal-dependent 4-oxalocrotonate decarboxylase (4-OD) generates 2-hydroxy-2,4-pentadienoate (**5a**) [9–11]. 4-OD functions in a complex with the next enzyme in the pathway, a metal-dependent vinylpyruvate hydratase (VPH) [7]. VPH catalyzes the addition of water to the C-4 position of **5a** to produce (*S*)-2-keto-4-hydroxypentanoate (**6a**) [9–11]. A retro-aldol cleavage of **6a** by pyruvate aldolase yields pyruvate and acetaldehyde (**7a**). Pyruvate aldolase is tightly coupled with an acetaldehyde dehydrogenase, which uses NAD^+^ and coenzyme A to produce acetyl-CoA (**8a**) [12]. Pyruvate and acetyl-CoA can then be funneled into the Krebs cycle.

**Scheme 2:**
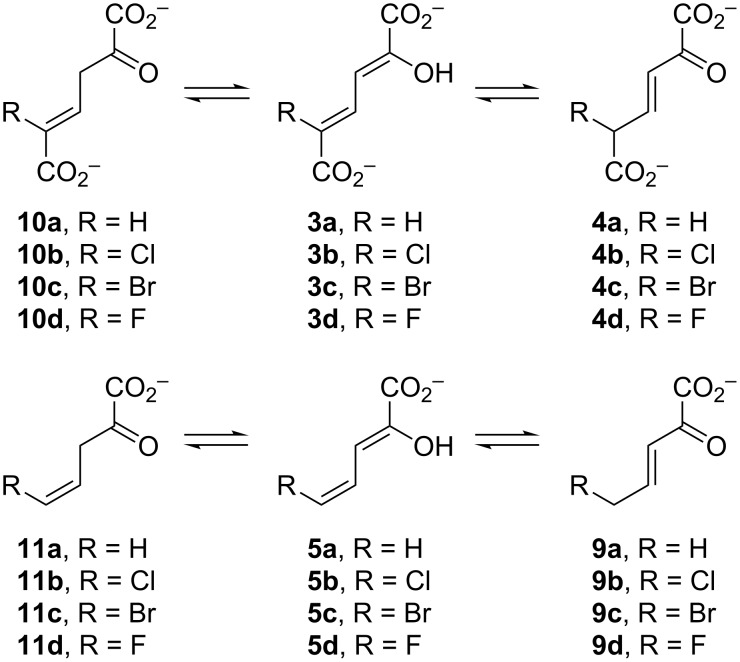
The ketonization of dienols **3** and **5**, to the corresponding α,β-unsaturated ketones (**4** and **9**, respectively) and the β,γ-unsaturated ketones (**10** and **11**, respectively).

Over the years, variations of this pathway have been reported that process halogenated catechols. One such pathway is found in *Comamonas* sp. strain CNB-1, which grows on 4-chloronitrobenzene as a sole carbon and energy source [3,13]. The reported pathway shows the chloro substituent at the C5 position to produce **3b**–**6b** (Scheme 1), but there is little chemical proof for the structures. In addition, if the proposed pathway in *C.* sp. strain CNB-1 follows that of the canonical one, then the actions of pyruvate aldolase and acetaldehyde dehydrogenase would produce 2-chloroacetaldehyde (**7b**) and 2-chloroacetyl CoA (**8b**), which are potential alkylating agents of these enzymes as well as other cellular proteins and DNA. This observation and the potential effects of the halogen on other enzyme-catalyzed steps in the pathway suggest that these enzymes might have evolved strategies to accommodate the halogen that mitigate potentially harmful consequences [4]. To explore these possibilities and the consequences of halogen substitution, a series of compounds were synthesized (**3b**–**d** and **5b**–**d**, Scheme 2) and a preliminary analysis of their properties carried out. The results are reported herein.

## Results and Discussion

### Synthesis of **3c**,**d** and **5c**,**d**

The 5-bromo- and 5-fluoro-2-hydroxymuconates (**3c** and **3d**) were successfully synthesized following the protocol used elsewhere to generate 5-chloro-2-hydroxymuconate (**3b**) [11]. These reactions combine the ethyl 2-halocrotonate with diethyl oxalate followed by alkaline hydrolysis and acidification. Subsequently, the 4*Z*-isomers of 5-bromo- and 5-fluoro-2-hydroxy-2,4-pentadienoates (**5c** and **5d**) were synthesized enzymatically (following the protocol for the 5-chloro derivative). The enzymatic synthesis relies on the actions of the 4-OT and 4-OD/E106QVPH (both from *P. putida* mt-2), which carry out ketonization and decarboxylation, respectively, of **3c** and **3d** [11]. (The 4-OD/E106QVPH retains full decarboxylase activity, but has very little hydratase activity [10].) The observation that this protocol generates the 4*Z*-isomers of **5b–d** indicates that the halogen does not affect the stereochemical outcome of the 4-OD-catalyzed reaction.

In the course of these experiments, it was observed that the generation of **5d** required the addition of 4-OT every minute over a 40 min period, whereas the generation of **5b**,**c** did not. Analysis of the reactions showed that all three resulted in the irreversible inactivation of 4-OT, but **5d** is the most potent. The full analysis and implications of these findings will be reported in the near future. However, these findings prompted us to investigate the 4-OT-catalyzed ketonization of these dienols, especially **5b–d**.

### Ketonization of **3a–d** by Pp and Lc 4-OTs

We have previously shown that *P. putida* mt-2 4-OT rapidly converts **3a** to β,γ-unsaturated ketone **10a** before a slower conversion to the α,β-unsaturated isomer **4a** [8]. Isomer **4a** is the thermodynamically stable one (≈80% at equilibrium) and the substrate for 4-OD, which is the next enzyme in the pathway (Scheme 1). The kinetic analysis for the ketonization of **3b–d** to **4b–d** is complicated by the much lower amounts of the α,β-unsaturated ketones present at equilibrium and the faster conversion of the dienols to the β,γ-unsaturated ketones [14]. Hence, we only examined the ketonization of the dienols to the β,γ-unsaturated ketones (following the loss of the λ_max_ associated with the dienol).

The steady-state kinetic parameters for the 4-OT-catalyzed ketonization of **3b–d** (to **10b–d**) were determined with the enzymes from *P. putida* mt-2 and *L. cholodnii* SP-6. 4-OT from *P. putida* mt-2 (designated Pp 4-OT) represents the canonical *meta*-fission pathway and 4-OT from *L. cholodnii* SP-6 (designated Lc 4-OT) represents the haloaromatic *meta*-fission pathway. The Lc 4-OT shows high similarity (78% identity and 87% similarity) with the one found in *Comamonas* sp. strain CNB-1, which is not available [11]. The Lc 4-OT is more distantly related to the Pp 4-OT (45% identity and 71% similarity).

The kinetic parameters are shown in Table 1 and Table 2. The kinetic parameters are comparable for two enzymes. For the Pp 4-OT, the *k*_cat_/*K*_m_ values for the fluoro derivative are higher (≈3–5-fold), mostly due to an increase in *k*_cat_. For the Lc 4-OT, the *k*_cat_/*K*_m_ values for the fluoro derivative are also higher, but not as high as those for the Pp enzyme. The kinetic parameters indicate that neither enzyme shows a preference for the halogenated compound and that this particular reaction is not affected by the presence of the halogen (although the ketonization of **3** to **10** is not the biological reaction). The “native” 4-OT activity (**3a** to **4a**) was measured for both enzymes and found to be comparable.

**Table 1:** Kinetic parameters for Pp 4-OT using **3a**–**d**.^a^

Reaction		*k*_cat_ (s^−1^)	*K*_m_ (μM)	*k*_cat_/*K*_m_ (M^−1^ s^−1^)

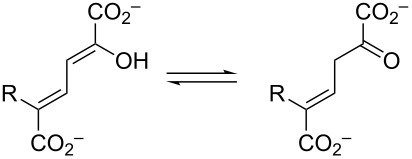				
**3b**: R = Cl	**10b**: R = Cl	220 ± 10	60 ± 10	3.8 ± 0.7 × 10^6^
**3c**: R = Br	**10c**: R = Br	210 ± 10	32 ± 3	6.6 ± 0.7 × 10^6^
**3d**: R = F	**10d**: R = F	630 ± 30	34 ± 4	1.9 ± 0.2 × 10^7^
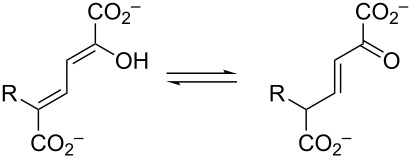				
**3a**: R = H	**4a**: R = H	2100 ± 100	100 ± 10	2.1 ± 0.2 × 10^7^

^a^The steady-state kinetic parameters were determined under the conditions described in the text. Errors are standard deviations.

**Table 2:** Kinetic parameters for Lc 4-OT using **3a**–**d**.^a^

Reaction		*k*_cat_ (s^−1^)	*K*_m_ (μM)	*k*_cat_/*K*_m_ (M^−1^ s^−1^)

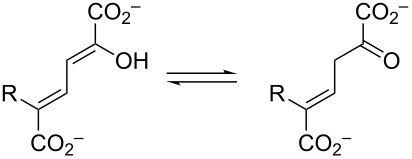				
**3b**: R = Cl	**10b**: R = Cl	350 ± 20	60 ± 10	5.8 ± 1.0 × 10^6^
**3c**: R = Br	**10c**: R = Br	420 ± 40	60 ± 10	7.0 ± 1.0 × 10^6^
**3d**: R = F	**10d**: R = F	420 ± 40	40 ± 10	1.1 ± 0.3 × 10^7^
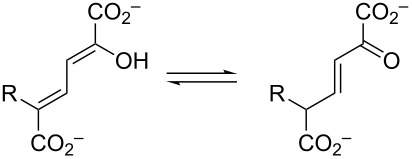				
**3a**: R = H	**4a**: R = H	2400 ± 100	170 ± 20	1.4 ± 0.2 × 10^7^

^a^The steady-state kinetic parameters were determined under the conditions described in the text. Errors are standard deviations.

### Composition of the equilibrium mixture for **5b–d**

Dienols **5b**–**d** were allowed to equilibrate in 100 mM Na_2_HPO_4_ buffer (final pH 6.8–7.2) in the presence of Pp 4-OT, and the identities of the components of the mixture were determined by ^1^H NMR spectroscopy. The ^1^H, ^13^C, and ^19^F NMR data are presented in Supporting Information File 1. The approximate percentages of the components were determined by integration and are summarized in Table 3 [15]. The highly electronegative fluoride substituent in **5d** prevents the detectable formation of the corresponding α,β-unsaturated ketone, **9d**.

**Table 3:** Equilibrium mixture of **5b–d**.

	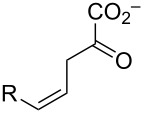	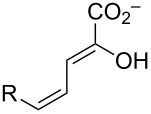	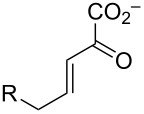

R = Cl	**11b** = 39% (+7% hydrate)^a^	**5b** = 15%	**9b** = 39%
R = Br	**11c** = 17% (+13% hydrate)^a^	**5c** = 9%	**9c** = 61%
R = F	**11d** = 54% (+22% hydrate)^a^	**5d** = 24%	N.D.^b^

^a^The hydrate of **11b–d** is present in varying amounts and depends on the halogen substitution. ^b^N.D. not detected.

### Ketonization of **3a–d** and **5a–d** by Pp and Lc 4-OT

Previous work has shown that 4-OT partitions a host of dienols to their β,γ- and α,β-unsaturated ketones [16–17]. The behavior of **5b**–**d** is consistent with these observations. The steady state kinetic parameters for the Pp 4-OT-catalyzed conversion of **5b**–**d** to the β,γ-unsaturated ketones (**11b**–**d**, respectively) and α,β-unsaturated ketones (**9b**,**c**, respectively) were determined, and compared to those for the non-halogenated species (**5a** to **11a** and **5a** to **9a**) (Table 4). For ketonization to the β,γ-unsaturated ketones, the *k*_cat_/*K*_m_ values are comparable ranging from 2.3 × 10^5^ (5-fluoro) to 1.8 × 10^6^ M^−1^ s^−1^ (5-bromo) where the 5-bromo species has the highest value (4-fold higher than the non-halogenated species). For ketonization to the α,β-unsaturated ketones, the *k*_cat_/*K*_m_ values are again comparable ranging from the 5.0 × 10^2^ M^−1^ s^−1^ (5-chloro) to 5.3 × 10^3^ M^−1^ s^−1^ (5-bromo). The α,β-unsaturated ketone **9d** (from **5d**) is not detectable (by UV or ^1^H NMR spectroscopy).

**Table 4:** Kinetic parameters for Pp 4-OT using **5a–d**.^a^

Reaction		*k*_cat_ (s^−1^)	*K*_m_ (μM)	*k*_cat_/*K*_m_ (M^−1^ s^−1^)

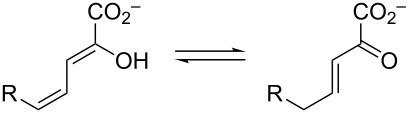				
**5a**: R = H	**9a**: R = H	2.9 ± 0.4	1600 ± 250	1.8 ± 0.4 × 10^3^
**5b**: R = Cl	**9b**: R = Cl	0.5 ± 0.1	1000 ± 250	5.0 ± 1.8 × 10^2^
**5c**: R = Br	**9c**: R = Br	1.7 ± 0.1	320 ± 30	5.3 ± 0.6 × 10^3^
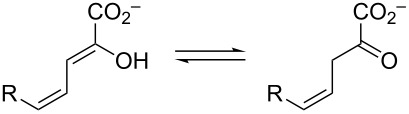				
**5a**: R = H	**11a**: R = H	150 ± 16	350 ± 50	4.3 ± 0.8 × 10^5^
**5b**: R = Cl	**11b**: R = Cl	320 ± 25	730 ± 87	4.4 ± 0.6 × 10^5^
**5c**: R = Br	**11c**: R = Br	340 ± 11	190 ± 12	1.8 ± 0.2 × 10^6^
**5d**: R = F	**11d**: R = F	110 ± 10	470 ± 56	2.3 ± 0.4 × 10^5^

^a^The steady-state kinetic parameters were determined under the conditions described in the text. Errors are standard deviations.

The steady state kinetic parameters for the Lc 4-OT-catalyzed conversion of **5b**–**d** to the β,γ-unsaturated ketones (**11b**–**d**, respectively) were determined and compared to those for the non-halogenated species (**5a** to **11a**, Table 5). In all cases, the *k*_cat_/*K*_m_ values are higher than that for **5a** ranging from 5.4–9.6-fold (for **5b** and **5c**, respectively). However, the values are lower than those determined for the Pp 4-OT-catalyzed reaction, ranging from 7.9–39-fold (for **5d** and **5c**, respectively).

**Table 5:** Kinetic parameters for Lc 4-OT using **5a–d^a^**.

Reaction		*k*_cat_ (s^−1^)	*K*_m_ (μM)	*k*_cat_/*K*_m_ (M^−1^ s^−1^)

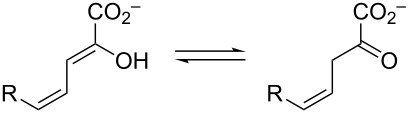				
**5a**: R = H	**11a**: R = H	2.0 ± 0.3	420 ± 89	4.8 ± 1.2 × 10^3^
**5b**: R = Cl	**11b**: R = Cl	36 ± 6	1400 ± 300	2.6 ± 0.7 × 10^4^
**5c**: R = Br	**11c**: R = Br	13.2 ± 0.5	290 ± 22	4.6 ± 0.4 × 10^4^
**5d**: R = F	**11d**: R = F	25 ± 2	850 ± 94	2.9 ± 0.4 × 10^4^

^a^The steady-state kinetic parameters were determined under the conditions described in the text. Errors are standard deviations.

The Lc 4-OT is not as efficient as the Pp 4-OT in generating the α,β-unsaturated ketones (**9a–c**) from the dienols (**5a**–**c**, respectively), and, in fact, requires a large quantity of enzyme. The high quantity of enzyme along with its overlapping absorbance precludes measurements at 232 nm and a determination of *k*_cat_/*K*_m_ values. To enable a rough comparison of the relative activities of the Pp and Lc 4-OTs, the absorbance at 232 nm was monitored for a longer time period with identical amounts of dienol (150 μM, Figure S1 in Supporting Information File 1). In the presence of 150 μM of **5a**, the Pp 4-OT (2 μM) generates 120 μM of **9a** in 16 min, whereas the Lc 4-OT (35 μM) generates at least 90 μM of **9a** in 20 min. (In both experiments, the A_232_ is outside the linear range so the amount of **9a** is likely being underestimated). For **5b**, the Pp 4-OT (2 µM) generates 33 μM of **9b** in 20 min, whereas the Lc 4-OT (2.3 μM) generates 11 μM of **9b** in 20 min. For **5c**, the Pp 4-OT (2 μM) generates 50 μM of **9c** in 5 min, whereas the Lc 4-OT (2.3 μM) generates 27 μM of **9c** in 20 min. At this concentration of dienol, Pp 4-OT is 20–30 ×, 3.5 × and 8.5 × faster at converting **5a**–**c** to **9a**–**c** than Lc 4-OT, respectively. In addition, the presence of the halogen accelerates the reaction.

## Conclusion

The 5-bromo and 5-fluoro-2-hydroxymuconates (**3c**,**d**) and 2-hydroxy-2,4-pentadienoates (**5c**,**d**) were synthesized following a protocol used to produce the 5-chloro derivatives [11]. The 4*Z*-isomers of **5c**,**d** are produced from the 4*Z*-isomers of **3c**,**d** using 4-OT and 4-OD/E106QVPH in the “one-pot” enzymatic synthesis. The stereochemical outcome of the 4-OD reaction is consistent with previous work indicating that it is not affected by the halogen [11]. In the course of the enzymatic synthesis, it was discovered that 4-OT is inactivated by **5b–d**, where **5d** is the most potent at these concentrations. If these downstream metabolites accumulate, the overall efficiency of the pathway might be affected. The non-enzymatic and 4-OT-catalyzed ketonization of **5b–d** (to the β,γ-unsaturated ketones and the α,β-isomers) was also examined as the presence of the different isomers could influence the inactivation process. Finally, it was determined that the Pp 4-OT is more efficient than the Lc 4-OT in the ketonization of **5b–d** to the β,γ-unsaturated ketones and the α,β-isomers. These results combined with our previous studies indicate that the presence of the halogen does not prevent enzymatic processing by 4-OT or 4-OD/VPH from either *P. putida* or *L. cholodnii*, and that the enzymes utilize similar mechanisms. The observation that **5b–d** inactivate 4-OT, will be reported in a forthcoming paper. Inactivation could affect the flux of halogenated intermediates through these pathways.

## Experimental

### Materials

Chemicals, biochemicals, buffers, solvents, and the components for Luria-Bertani (LB) media were obtained from sources reported elsewhere [11]. The synthesis of 2-hydroxymuconate (**3a**) [8], 2-hydroxy-2,4-pentadienoate (**5a**) [10], 5-chloro-2-hydroxymuconate (4*Z***-3b**) [11], and (4*Z*)-5-chloro-2-hydroxy-2,4-pentadienoate (**5b**) [11], and ethyl 2-fluorocrotonate [18] are reported in the indicated references. The Phenyl Sepharose 6 Fast Flow resin and the pre-packed PD-10 Sephadex G-25 columns were obtained from GE Healthcare (Piscataway, NJ). The Econo-Column chromatography columns were obtained from Bio-Rad Laboratories, Inc. (Hercules, CA). 4-OT, 4-OD/VPH, and 4-OD/E106QVPH (all from *P. putida* mt-2) were purified by procedures reported elsewhere with minor modifications [10–11,19–20]. The YM-3 ultrafiltration membranes and centrifugal microconcentrators were obtained from Millipore (Billerica, MA). Activities were determined using previously described assays [10–11,19–20]. The plasmids containing the genes for 4-OT and 4-OD/VPH from *P. putida* mt-2 and *L. cholodnii* SP-6, and 4-OD/E106QVPH from *P. putida* mt-2 were constructed as reported elsewhere [10–11]. Proteins were expressed as described.

### General methods

Mass spectral data were obtained on an LCQ electrospray ion-trap mass spectrometer (Thermo, San Jose, CA) in the ICMB Protein and Metabolite core facility. The samples were prepared as described previously [21]. Kinetic data were obtained at 24 °C on an Agilent 8453 diode-array spectrophotometer. 4-OT was assayed using **3a**, as previously reported [19–20]. Protein concentrations were determined by the Waddell method [22]. 4-OT was analyzed using tricine SDS-PAGE on 15% gels [23]. All other proteins were analyzed using TRIS-glycine SDS-PAGE on 12% gels [24]. Gels were run on a Bio-Rad Mini-Protean II gel electrophoresis apparatus.

### Purification of 4-OT from L. cholodnii SP-6

In a typical procedure, frozen cells (25 g) were thawed on ice and suspended in 120 mL of 20 mM NaH_2_PO_4_ buffer (pH 7.3). Lysozyme was added to the cell suspension to a final concentration of 5 mg/mL and the resulting mixture was allowed to incubate for 30 min before sonication (20% duty cycle on 5 s cycle for 25 min using a Heat Systems W-385 sonicator equipped with a 0.5-in. tapped horn delivering approximately 330 W/pulse). After sonication, the lysed cell mixture was centrifuged (17500*g* for 45 min) and the pellet discarded. The supernatant was placed in a boiling water bath for 15 min, centrifuged (17500*g* for 30 min), and the pellet was discarded. The supernatant was put on ice, and with slow stirring, was brought up to 75% (NH_4_)_2_SO_4_ saturation (3.6 M). After stirring for 30 min, the cloudy solution was centrifuged (17500*g* for 30 min) and the pellet was discarded. The supernatant was loaded onto a hand-packed Phenyl-Sepharose column (15 mL of resin) equilibrated with 20 mM NaH_2_PO_4_ buffer (pH 7.3) made 2 M in (NH_4_)_2_SO_4_. After loading the solution, the column was washed with the equilibrating buffer (150 mL). Protein was eluted using a linear gradient [2–0 M (NH_4_)_2_SO_4_)]. Fractions (≈2 mL) were collected and pooled by their 4-OT activity. The protein sometimes appears as a series of bands (multiples of the monomer mass) by SDS-PAGE where no one band corresponds to the monomer mass (6915 Da). However, if the protein is exchanged into 20 mM HEPES buffer (pH 7.3), loaded onto a HiLoad 16/60 Superdex 75 prep grade column (120 mL resin), equilibrated, and eluted with the same buffer (at 0.5 mL/min), a single band of the correct size is observed. The enzyme elutes at 104 mL, which is consistent with the monomer mass. Pooled fractions were concentrated and exchanged into 20 mM KH_2_PO_4_ buffer (pH 7.3) using an Amicon Ultra filter unit (3K membrane). This procedure typically yields ≈8 mg of 4-OT estimated to be ≈95% pure. A sample was analyzed by electrospray ionization mass spectrometry (ESIMS) to verify the molecular mass (6916 Da). In addition to this species (corresponding to the intact enzyme without the *N*-formylmethionine), there are five additional signals at 6475 Da, 6606 Da, 6634 Da, 7046 Da, and 7076 Da. These signals correspond to enzyme without the four C-terminal amino acids, the same enzyme with an N-terminal methionine, the same enzyme with an *N*-formylmethionine, the intact enzyme with an N-terminal methionine, and the intact enzyme with an *N*-formylmethionine [25].

### Synthesis of ethyl 2-fluorocrotonate and ethyl 2-bromocrotonate

Ethyl 2-fluorocrotonate was synthesized following published procedures [18]. Ethyl 2-bromocrotonate was synthesized following the protocol for the synthesis of ethyl 2-chlorocrotonate except that liquid bromine was added dropwise under argon in place of chlorine gas [11]. Accordingly, ethyl crotonate (15 g) was dissolved in CH_2_Cl_2_ (100 mL), chilled in an ice bath, and a solution of bromine (21 g) dissolved in CH_2_Cl_2_ (150 mL) was added over a period of 30 min. The solution was allowed to come to ambient temperature overnight. The solvent was removed under reduced pressure and the residue was distilled (3 torr, 79–81 °C) to leave a pale yellow liquid (29.7 g). The ^1^H and ^13^C NMR analysis indicated that the product formed was ethyl 2,3-dibromobutyrate. Subsequently, ethyl 2,3-dibromobutyrate was converted to ethyl 2-bromocrotonate following the procedure used to convert ethyl 2,3-dichlorobutyrate to ethyl 2-chlorocrotonate [11].

### Syntheses of 5-(halo)-2-hydroxymuconate (**3c**,**d**)

The syntheses of the 4*Z*-isomers of **3c** and **3d** (5-bromo- and 5-fluoro-, respectively) were based on the synthesis of (4*Z*)**-3b**, which followed the procedure used to produce 2-hydroxymuconate (**3a**) [8,11]. Sodium ethoxide was generated by allowing sodium metal (1 equiv, 26.2 and 17.3 g for **3c** and **3d**, respectively) to react completely with ethanol (50 mL), followed by the addition of toluene (300 mL) to the stirring mixture. The resulting solution was distilled (to remove water and ethanol) under an argon atmosphere until the temperature climbed above 80 °C. The anhydrous sodium ethoxide was chilled in an ice bath and diethyl oxalate (1 equiv, 6.5 g and 4.3 g for **3c** and **3d**, respectively) was added, followed by the addition of ethyl 2-bromo- or ethyl 2-fluorocrotonate [1 equiv, 6.7 g (42.4 mmol) and 4.4 g (27.9 mmol) for **3c** and **3d**, respectively]. The ethyl 2-halocrotonates consisted of an *E*/*Z* mixture with a predominance of the *E*-isomer. The reaction mixture was allowed to warm to ambient temperature. After being stirred at room temperature for 72 h, the mixture was filtered and the precipitate washed with ether until the filtrate was clear. The precipitate was air-dried to yield the crude sodium salt of the diethyl ester of **3c** and **3d**, respectively. The free acid was prepared by alkaline hydrolysis of the diethyl ester and subsequent acidification as follows. The diethyl ester was suspended in water and chilled in an ice bath. Subsequently, a solution of 1 M NaOH (2.5 equiv) was added and the mixture stirred at ambient temperature for 16 h. The reaction mixture was filtered and the filtrate was adjusted to pH 1 by the addition of concentrated HCl. The precipitate was collected by filtration and crystallized in ethyl acetate (**3c**: 1.18 g and **3d**: 1.56 g). The ^1^H, ^13^C, and ^19^F NMR data are presented in Supporting Information File 1.

### Syntheses of 5-(halo)-2-hydroxy-2,4-pentadienoate (**5c**,**d**)

The preparation of the 4*Z*-isomers of **5c** and **5d** was adapted from published procedures [10–11]. The diacid (200 mg, **3c** or **3d**) is combined with ≈20 mM Na_2_HPO_4_ buffer (25 mL), and the pH was adjusted to 6.5–7.2 by the addition of aliquots of a 5 M NaOH solution. Adjustment of the pH results in the dissolution of the diacid. An aliquot (≈125 μL) of a 1 M MgCl_2_ solution (for a final concentration of 3–5 mM) was then added to a suspension. For the preparation of **5c**, 4-OT is added to the diacid mixture and the mixture was incubated for 10 min. Subsequently, a sufficient quantity of 4-OD/E106QVPH (from *P. putida* mt-2) was added so that the reaction was complete within 20 min (monitored by UV spectroscopy). For the preparation of **5d**, an aliquot of 4-OT was added to the diacid mixture (which already contained 4-OD/E106QVPH and MgCl_2_) every min up to a 40 min period. The pH of both mixtures was adjusted to 1.8–2.0. The solution was extracted with ethyl acetate (3×), and the organic layers were pooled, dried over anhydrous Na_2_SO_4_, filtered, and evaporated to dryness at room temperature. The resulting solid was dissolved in CH_2_Cl_2_, and filtered through a nylon filter to remove the residual diacid. The organic layer was collected and evaporated to dryness at room temperature. The solid was dissolved in methanol, and the solution was passed through a nylon filter, collected, and diluted with ethyl acetate to azeotrope any water that is present in the methanol. The resulting solution was evaporated to dryness under reduced pressure at room temperature to yield the monoacid (**5c** or **5d**). Titration with hexanes yields a sticky yellow solid (≈100 mg). The compounds are stored at −20 °C. The ^1^H, ^13^C, and ^19^F NMR data are presented in Supporting Information File 1.

### Equilibrium composition mixtures of **5b–d**

In separate test tubes, **5b**–**d** (4 mg) was dissolved in dimethyl sulfoxide (DMSO)-*d*_6_ (30 μL). The individual mixtures were then added to 100 mM Na_2_HPO_4_ buffer (600 μL, pH ≈9). The final pH ranged from 6.8–7.2. The Pp 4-OT (2 μL of a 43 mg/mL solution) was added to the mixture and the resulting mixture was placed in an NMR tube. The reactions were monitored by recording scans every 3 min until equilibrium had been reached (within 10 min). The spectra for the final mixtures were similar to those recorded for the samples that were allowed to equilibrate in buffer overnight. The approximate amounts of product in the mixtures were determined by integration of the signals, as described previously [15,26]. The C3 methylene protons show signals in the range of 3.39–3.53 ppm (2.41–2.53 ppm if hydrated) and the C5 methylene protons show signals in the range of 4.07–4.16 ppm. The ^1^H NMR data are presented in Supporting Information File 1.

### 4-OT-catalyzed ketonization of **5a–d**

The 4-OT-catalyzed ketonization of **5a**–**d** (Scheme 2) [27–28] was examined by following the decrease in absorbance at 280 nm (ε = 8700 M^−1^ cm^−1^), 304 nm (ε = 1300 M^−1^ cm^−1^), 304 nm (ε = 2300 M^−1^ cm^−1^), and 284 nm (ε = 4600 M^−1^ cm^−1^), respectively [9,22–23]. The decrease in absorbance corresponds to the ketonization of the dienol to the β,γ-unsaturated ketones (**11a–d**, respectively). These wavelengths and extinction coefficients were used to increase the concentration range and keep the collected data in the linear range of absorbance. The λ_max_ values for **5a–d** are 265 nm (ε = 12700 M^−1^ cm^−1^), 278 nm (ε = 14500 M^−1^ cm^−1^), 281 nm (ε = 15100 M^−1^ cm^−1^), and 266 nm (ε = 9400 M^−1^ cm^−1^), respectively. The reactions were carried out in 20 mM Na_2_HPO_4_ buffer (1 mL, pH 7.3) containing Pp 4-OT (500 nM for **5a** and 66 nM for **5b–d**) or Lc 4-OT (3200 nM for **5a**, 500 nM for **5b** and **5d**, 1400 nM for **5c**). Assays were initiated by addition of varying amounts of **5a–d** (20–160 μM, 40–600 μM, 20–400 μM, and 20–300 μM, respectively) from stock solutions made up in ethanol (20, 40, 20, and 20 mM, respectively). The rapid non-enzymatic rates for **5a**–**c** were subtracted from the observed enzymatic rates. The non-enzymatic rate for **5d** was negligible. Data were collected every 0.5 s and the initial slope (resulting in the first 10 s of the reaction) was fit to a zero-order equation. The initial rates were determined in triplicate, averaged, plotted versus initial substrate concentration, and fit to determine *k*_cat_ and *K*_m_. Nonlinear regression data analysis was performed using Mathematica (Wolfram Research, Inc., Mathematica, Version 8.0, Champaign, IL 2010).

The 4-OT-catalyzed conversion of dienols **5a**–**c** to their respective α,β-unsaturated ketones **9a**–**c** (Scheme 2) was measured by following the increase in absorbance at 232 nm [9–10,14,16]. (There is no measureable increase in the absorbance at 232 nm for the reaction of 4-OT and **5d**, suggesting no detectable formation of **9d**.) Extinction coefficients were determined for **9b** and **9c** following the protocol used to determine the extinction coefficient for **9a** [9]. Briefly, the absorbance of a known concentration of dienol (**5b** and **5c**) initially made up in ethanol and added to 20 mM NaH_2_PO_4_ buffer (pH 7.3) was determined at 232 nm (ε = 1700 M^−1^ cm^−1^ and 1790 M^−1^ cm^−1^, respectively). The solutions were then allowed to equilibrate in 20 mM NaH_2_PO_4_ buffer (pH 7.3). The final absorbance at 232 nm was then corrected for the remaining dienol at equilibrium (15% and 9% for **5b** and **5c**, respectively) as well as the amount of conjugated ketone at equilibrium (39% and 61% for **9b** and **9c**, respectively). The ketonization reactions were carried out in 10 mM NaH_2_PO_4_ buffer (1 mL, pH 7.3) containing 4 μM of Pp 4-OT. Assays were initiated by the addition of varying amounts of **5a**, **5b**, or **5c** (20–200 μΜ) from stock solutions made up in ethanol (20 mM). The increase in absorbance at 232 nm was assumed to be entirely due to the formation of the conjugated ketone (ε = 5990 M^−1^ cm^−1^, 11500 M^−1^ cm^−1^, and 9400 M^−1^ cm^−1^, respectively). Data were collected every 0.5 s and the initial slope (5–20 s of the reaction) was fit to a zero-order equation. In the first 5 s of the reaction, the absorbance at 232 nm was still decreasing. Kinetic parameters were determined as described above.

The Lc 4-OT conversion of **5a**–**c** to the respective α,β-unsaturated ketones **9a**–**c** was compared to the Pp 4-OT-catalyzed conversion by monitoring the spectral changes over 20 min. Aliquots of **5a**–**c**, made up as 20 mM stock solutions in ethanol, were added to 20 mM NaH_2_PO_4_ buffer (1 mL, pH 7.3) with Pp 4-OT (2 μM) or Lc 4-OT (35 μM for **5a** or 2.3 μM for **5b**,**c**).

The 4-OT-catalyzed ketonization of **3b**–**d** (Scheme 2) was examined by following the decrease in absorbance at 284 nm (ε = 9200 M^−1^ cm^−1^), 327 nm (ε = 8400 M^−1^ cm^−1^), and 272 nm (ε = 11300 M^−1^ cm^−1^), respectively. The decrease in absorbance corresponds to the ketonization of the dienol to the β,γ-unsaturated ketones (**3b**–**d** to **10b**–**d**, respectively). These wavelengths and extinction coefficients were used to increase the concentration range and keep the collected data in the linear range of absorbance. The λ_max_ values for **3b**–**d** are 304 nm (ε = 14700 M^−1^ cm^−1^), 307 nm (ε = 15100 M^−1^ cm^−1^), and 292 nm (ε = 18300 M^−1^ cm^−1^), respectively. The reactions were carried out in 10 mM K_2_HPO_4_ buffer (1 mL, pH 7.3) containing Pp 4-OT (12 nM for **3b** and **3c**, 1 nM for **3d**) or Lc 4-OT (8.75 nM for **3b**, 10.5 nM for **3c**, 1.75 nM for **3d**). Assays were initiated by addition of varying amounts of **3b**–**d** (15–120 μM, 16–125 μM, and 15–120 μM, respectively) from stock solutions made up in ethanol (20, 6.25, and 20 mM, respectively). The rapid non-enzymatic rates of **3b**–**d** were subtracted from the observed enzymatic rates. Data were collected every 0.5 s and the initial slope (resulting in the first 6 s of the reaction for **3b** and **3c**, the first 10 s of the reaction for **3d**) was fit to a zero-order equation. Kinetic parameters were determined as described above.

### The 4-OT-catalyzed ketonization of **3a** to **4a**

The Pp and Lc 4-OT-catalyzed conversion of **3a** to the α,β-unsaturated ketone, **4a** (Scheme 2) was measured by following the increase in absorbance at 236 nm [8]. The reactions were carried out in 10 mM KH_2_PO_4_ buffer (1 mL, pH 7.3) containing 4 nM of Pp 4-OT or 1.75 nM of Lc 4-OT. Assays were initiated by the addition of varying amounts of **3a** (20–200 μΜ) from a stock solution made up in ethanol (20 mM). The increase in absorbance at 236 nm was assumed to be entirely due to the formation of the conjugated ketone (ε = 6580 M^−1^ cm^−1^). Data were collected every 0.5 s and the initial slope (first 6 s of the reaction) was fit to a zero-order equation. Kinetic parameters were determined as described above. Both enzymes catalyze the conversion of **3b–d** (to **4b–d**), as determined by visual inspection, but kinetic parameters were not obtained.

## Supporting Information

File 1 Analytical data.
