# Supplementary material for: Synthesis and enzymatic ketonization of the 5-(halo)-2-hydroxymuconates and 5-(halo)-2-hydroxy-2,4-pentadienoates
Source: Beilstein J Org Chem. 2017 May 26;13:1022–31. doi: 10.3762/bjoc.13.101 (PMC5480330; doi:10.3762/bjoc.13.101)
Supplement: File 1 — Analytical data. [file Beilstein_J_Org_Chem-13-1022-s001.pdf]

Supporting Information File 1

for

Synthesis and enzymatic ketonization of the 5-(halo)-2-hydroxymuconates and 5-(halo)-2-hydroxy-2,4-pentadienoates

Tyler M. M. Stack[1], William H. Johnson Jr.[2] and Christian P. Whitman[*2]

Address: [1]Department of Molecular Biosciences, College of Natural Sciences, 1 University Station, University of Texas, Austin, TX 78712, USA and [2]Division of Chemical Biology and Medicinal Chemistry, College of Pharmacy, 1 University Station, University of Texas, Austin, TX 78712, USA

Email: Christian P. Whitman[*] - whitman@austin.utexas.edu

[*]Corresponding author

Analytical data

Table of Contents

General NMR information S2

^{1}H NMR, ^{13}C NMR, and ^{19}F NMR spectral data for 5-bromo-2-hydroxymuconate (3c) and 5-fluoro-2-hydroxymuconate (3d) S2

^{1}H NMR, ^{13}C NMR, and ^{19}F NMR spectral data for 5-bromo-2-hydroxypentadienoate (5c) and 5-fluoro-2-hydroxypentadienoate (5d) S2

^{1}H NMR spectral data for equilibrium products of the 5-halo-2-hydroxypentadienoates in sodium phosphate buffer pH 7. .... S3

Figure S1: Time-dependent enzymatic conversions of 5-halo-2-hydroxypentadienoates to the corresponding α,β-unsaturated ketones. S4

General NMR information

Nuclear magnetic resonance spectra (^{1}H NMR, ^{13}C NMR, and ^{19}F NMR) were recorded on a Varian Unity INOVA-500 or -300 MHz spectrometer, or on an Agilent 400 MHz spectrometer, and reported in parts per million (ppm) referenced to the residual protio solvent signal as an internal standard.

^{1}H NMR, ^{13}C NMR, and ^{19}F NMR spectral data for 5-bromo-2-hydroxymuconate (3c) and 5-fluoro-2-hydroxymuconate (3d)

(4*Z*)-**3c**: ^{1}H NMR (CD_3OD, 300 MHz) δ 6.46 (1H, d, *J* = 11.7 Hz, H3), 8.14 (1H, d, *J* = 11.7 Hz, H4) ppm; ^{13}C NMR (CD_3OD, 75 MHz) δ 106.6 (C3), 115.1 (C4), 135.1 (C5), 148.9 (C2), 164.7 (C6), 165.1 (C1) ppm. (4*Z*)-**3d**: ^{1}H NMR (DMSO-d_6, 600 MHz) δ 6.20 (1H, d, J = 12.1 Hz, H3), 6.94 (1H, dd, J_{HF} = 32.1 Hz, H4) ppm; ^{13}C NMR (DMSO-d_6, 100.5 MHz) δ 100.1 (C3), 111.6, 111.7 (d, C4), 145.3, 147.9 (d, $J_{C,F}$ = 263 Hz, C5), 146.9 (C2), 161.2, 161.6 (d, C6), 164.5 (C1) ppm. ^{19}F NMR (DMSO-d_6, 564 MHz) δ -128.7 (d, $J_{H,F}$ = 32.1 Hz) ppm. The coupling constant (32.1 Hz) for the C4 proton and C5 fluoride indicates a *trans*-like relationship. Hence, the procedure affords (4*Z*)-**3d**. It can be inferred based on this observation that (4*Z*)-**3c** was also generated.

^{1}H NMR, ^{13}C NMR, and ^{19}F NMR spectral data for 5-bromo-2-hydroxypentadienoate (5c) and 5-fluoro-2-hydroxypentadienoate (5d)

(4*Z*)-**5c**: ^{1}H NMR (CD_3OD, 400 MHz) δ 6.36 (1H, dd, *J* = 1.2 Hz, 7.2 Hz, H3), 6.42 (1H, dd, *J* =1.2 Hz, 10.8 Hz, H5), 7.08 (1H, dd, *J* = 7.2 Hz, 10.8 Hz, H4) ppm; ^{13}C NMR (CD_3OD, 75 MHz) δ 107.7 (C3), 110.5 (C4), 127.6 (C5), 145.1 (C2), 167.7 (C1) ppm. (4*Z*)-**5d**: ^{1}H NMR (DMSO-d_6, 600 MHz) δ 5.85 (1H, ddd, *J* = 4.4 Hz, 11.7 Hz, J_{HF} = 43.1 Hz, H4), 6.20 (1H, d, *J* =

11.7 Hz, H3), 6.77 (1H, dd, J = 4.1 Hz, J_{HF} = 84.3 Hz, H5), 9.07 (1H, s, H2), 13.04 (1H, br, H1) ppm; ^{13}C NMR (DMSO-d_6, 75 MHz) δ 100.9 (d, $J_{C,F}$ = 5.9 Hz, C3), 105.1 (d, $J_{C,F}$ = 1.2 Hz, C4), 142.1 (d, $J_{C,F}$ = 4.7 Hz, C2), 149.5 (d, $J_{C,F}$ = 267 Hz, C5), 165.3 (C1) ppm. ^{19}F NMR (DMSO-d_6, 282 MHz) δ -124.3 (dd, $J_{H,F}$ = 42.3 Hz, 83.5 Hz) ppm. Both the ^{1}H and ^{13}C NMR assignments were verified by Heteronuclear Single Quantum Correlation (HSQC) experiments.

^{1}H NMR spectral data for equilibrium products of the 5-halo-2-hydroxypentadienoates in sodium phosphate buffer pH 7.

(4*Z*)-**5b**: ^{1}H NMR (100 mM NaH_2PO_4 buffer in H_2O, pH 7, 5% DMSO-d_6, 600 MHz) δ 6.00 (1H, dd, J = 0.9 Hz, 7.1 Hz, H3), 6.52 (1H, dd, J = 7.1 Hz, 11.2 Hz, H4), 6.80 (1H, not resolved, H5) ppm; (4*Z*)-**11b**: δ 3.53 (2H, dd, J = 1.4 Hz, 7.0 Hz, H3), 5.83 (1H, dt, J = 7.0 Hz, 7.0 Hz, H4), 6.20 (1H, dt, J = 7.0 Hz, 1.4 Hz, H5) ppm; (3*E*)-**9b**: δ 4.18 (2H, dd, J = 1.6 Hz, 5.8 Hz, H5), 6.32 (1H, dt, J = 1.6 Hz, 15.8 Hz, H3), 6.86 (1H, dt, J = 5.8 Hz, 15.8 Hz, H4) ppm; (4*Z*)-**5c**: ^{1}H NMR (100 mM NaH_2PO_4 buffer in H_2O, pH 7, 5% DMSO-d_6, 600 MHz) δ 6.13 (1H, dd, J = 0.9 Hz, 10.9 Hz, H3), 6.16 (1H, dd, J = 0.9 Hz, 11.2 Hz, H5), 6.83 (1H, dd, J = 7.1 Hz, 10.9 Hz, H4) ppm; (4*Z*)-**11c**: δ 3.52 (2H, dd, J = 1.3 Hz, 6.9 Hz, H3), 6.17 (1H, d, J = 7.0 Hz, H5), 6.37 (1H, dt, J = 7.0 Hz, 1.3 Hz, H4) ppm; (3*E*)-**9c**: δ 4.02 (2H, dd, J = 1.1 Hz, 7.2 Hz, H5), 6.26 (1H, dt, J = 1.1 Hz, 15.8 Hz, H3), 6.87 (1H, dt, J = 7.2 Hz, 15.8 Hz, H4) ppm; ^{1}H NMR (100 mM NaH_2PO_4 in H_2O, pH 7, 5% DMSO-d_6, 600 MHz) (4*Z*)-**5d**: δ 5.64 (1H, ddd, J = 4.6 Hz, 11.6 Hz, J_{HF} = 42.2 Hz, H4), 6.09 (1H, d, J = 11.6 Hz, H3), 6.45 (1H, dd, J = 4.6 Hz, J_{HF} = 83.9 Hz, H5) ppm; (4*Z*)-**11d**: δ 3.39 (2H, dt, J = 1.3 Hz, 7.4 Hz, H3), 4.85 (1H, m, H4), 6.53 (1H, dt, J = 4.7 Hz, 1.3 Hz, J_{HF} = 85 Hz, H5) ppm.

In addition, the hydrates of **11b** (7%), **11c** (13%), and **11d** (22%) were observed. The ^{1}H NMR signals for the three hydrates are as follows. (4*Z*)-**11b** (hydrate): δ 2.53 (2H, dd, J = 1.7

Hz, 7.0 Hz, H3), 5.63 (1H, dt, J = 7.1 Hz, 7.1 Hz, H4), 6.05 (1H, dt, J = 1.7 Hz, 7.1 Hz, H5) ppm; (4*Z*)-**11c** (hydrate): δ 2.51 (2H, dd, J = 1.6 Hz, 6.9 Hz, H3), 5.97 (1H, dt, J = 6.9 Hz, 7.0 Hz, H4), 6.20 (1H, dt, J = 1.6 Hz, 7.0 Hz, H5) ppm; (4*Z*)-**11d** (hydrate): δ 2.41 (2H, dt, J = 1.4 Hz, 7.7 Hz, H3), 6.42 (1H, dt, J = 1.4 Hz, 4.78 Hz, J_{HF} = 85.9 Hz, H5) ppm. The signal corresponding to H4 appears to be obscured by the water signal.

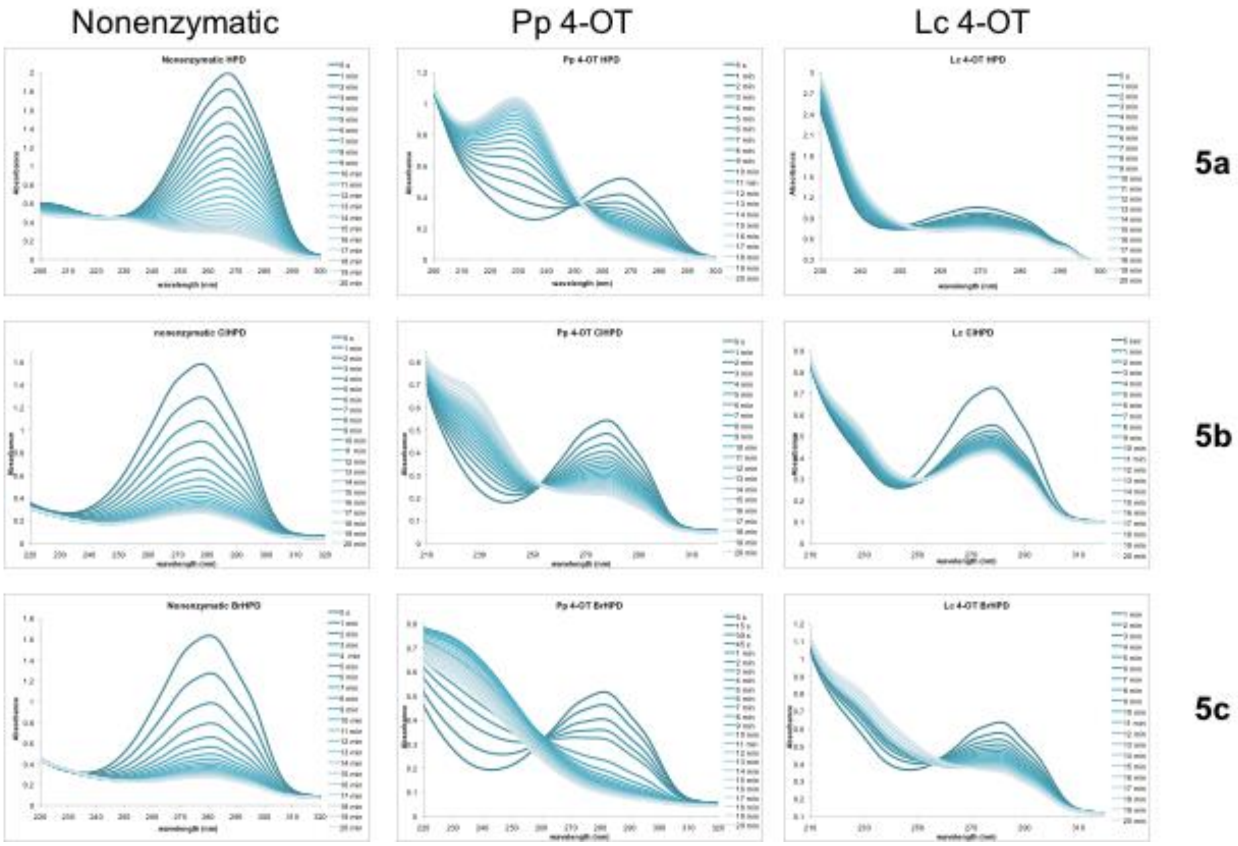

Figure S1: Time-dependent enzymatic conversions of 5-halo-2-hydroxypentadienoates to the corresponding α,β-unsaturated ketones.

Comparison of non-enzymatic and enzymatic UV changes of **5a**, **5b**, and **5c**. Each UV trace was obtained by monitoring 150 µM of **5a**, **5b**, or **5c** in 20 mM sodium phosphate buffer, pH 7.3. The UV progression was monitored for 20 min, with one trace displayed per minute. The UV traces

are shown as fading from blue to gray, with the initial trace (5 s) is the darkest trace, and the final 20 min trace is the lightest trace. In the traces containing Pp 4-OT, 2 μM (monomer mass) was used, while in the traces containing Lc 4-OT, 2.3 μM (monomer mass) was used except in the mixture containing **5a**, where 35 μM (monomer mass) was used. The abbreviations HPD, ClHPD, and BrHPD refer to **5a**, **5b**, and **5c**, respectively.
